# Rapid detection of H5 subtype avian influenza virus using CRISPR Cas13a based-lateral flow dipstick

**DOI:** 10.3389/fmicb.2023.1283210

**Published:** 2023-11-29

**Authors:** Yang Li, Jiajing Shang, Juan Luo, Fuyou Zhang, Ge Meng, Yingjie Feng, Wenming Jiang, Xiaohui Yu, Chunran Deng, Guanhui Liu, Hualei Liu

**Affiliations:** ^1^China Animal Health and Epidemiology Center, Qingdao, China; ^2^School of Life Sciences and Food Engineering, Hebei University of Engineering, Handan, China

**Keywords:** RAA, CRISPR Cas13a, lateral flow dipstick, H5-AIV, rapid diagnosis

## Abstract

Due to its high mortality rate, highly pathogenic avian influenza (HPAI), a notifiable animal illness designated by the World Organisation for Animal Health (WOAH), has caused enormous financial losses to the poultry sector. The H5 subtype of avian influenza virus (H5-AIV) is regarded as the most common highly pathogenic avian influenza virus (HPAIV) that threatens public health and safety. Virus isolation and reverse transcription quantitative PCR (RT-qPCR) are usually used to detect H5-AIV and are important for the timely diagnosis and control of H5-AIV. However, these methods are time-consuming and require a significant amount of effort. In this study, we established a recombinase-aided amplification (RAA) combined with CRISPR-Cas13a and lateral flow dipstick (LFD) assay for the detection of H5-AIV. The results showed that the process can be completed within 40 min at 37°C. The method had a detection limit of 0.1 copy/μL, which was comparable to the RT-qPCR. There was no cross-reactivity with H3-AIV, H7-AIV, H9-AIV, H10-AIV, IBV, NDV, RVA and DAstV. The kappa value of RT-RAA-Cas13a-LFD and RT-qPCR in 380 clinical samples was 0.89 (*κ*>0.75). In conclusion, we established a convenient, efficient and accurate method to detect H5-AIV, and the results can be visualized and interpreted using LFD, which can be adapted to the needs of grassroots laboratories and field-deployable assays. This approach provides a new perspective for clinical H5-AIV diagnosis and has great potential for application in clinical quarantine of the poultry farming.

## Introduction

Due to its high mortality rate, highly pathogenic avian influenza (HPAI), a notifiable animal illness designated by the World Organization for Animal Health (WOAH), has caused enormous financial losses to the poultry sector.^1^ As one of the HPAIV, H5-AIV can be described as a positive-sense single-stranded, non-enveloped RNA virus. It belongs to the genus of influenza viruses in the Orthomyxoviridae family (Nuñez and Ross, 2019). A serious threat to the world’s poultry business and public health has emerged in recent years as a result of the HPAI viruses of clade 2.3.4.4 H5Nx spreading to wild and domestic birds worldwide. These viruses cause annual mortality rates in wild birds and outbreaks in poultry in numerous European nations (Liang et al., 2022; Cha et al., 2023). In 2022, outbreaks of H5N1-AIV in poultry and wild birds were reported in more than 60 countries worldwide, with more than 131 million poultry killed or culled. The clinical symptoms caused by H5-AIV include appetite loss, depression, ataxia, decreased egg production, and in severe cases, a 100% incidence rate of death. According to the World Health Organization (WHO), nearly 900 cases of human infection with H5N1 have been reported worldwide over the past 20 years, with more than 450 deaths. The rapid mutation of H5-AIV and its wide range of hosts make it difficult to prevent and control the infection. Therefore, there is an urgent need for early detection and prevention of H5-AIV to control the spread of the virus and to reduce the economic losses of the poultry farming industry. Currently, the main methods for detecting H5-AIV include traditional virus isolation (Krauss et al., 2012), polymerase chain reaction (PCR) (Wei et al., 2006), and real-time quantitative PCR (RT-qPCR) (Le et al., 2020). However, these methods are time-consuming, require specialized personnel and expensive instrumentation, and cannot fulfill the requirement for rapid H5-AIV detection. Therefore, it is imperative to establish a new assay for rapid and accurate detection of H5-AIV for primary veterinary medicine and grassroots laboratories.

As an adaptable antiviral immune system in prokaryotes, *Leptotrichia wadei* (Lwa) clustered regularly interspaced short palindromic repeats (CRISPR/Cas13a) can be found in most bacteria (Koonin and Makarova, 2009). Due to its capacity for trans-cleavage to precisely identify and cleave particular nucleic acid targets, it has also been used as a potent and useful nucleic acid detection assay (Koonin and Makarova, 2009; Chang et al., 2020; Yang et al., 2023). The ability of Cas13a to identify the presence of RNA targets by CRISPR RNA (crRNA) and collateral cleavage activity was demonstrated in 2016 (Abudayyeh et al., 2016). When crRNA and the target single-stranded RNA are paired in a complementary manner, Cas13a binds to form a complex and undergoes a conformational change that activates non-specific RNase activity, allowing it to target cleavable accessible single-stranded RNA (ssRNA) (Knott et al., 2017; Yao et al., 2021), and the results can be assayed with a FAM-BHQ labeled reporter RNA for fluorescent readout or FAM-Biotin labeled reporter RNA for lateral flow dipstick (LFD) (Myhrvold et al., 2018). Recombinase-aided amplification (RAA) represents a new thermostatic nucleic acid amplification technology that allows for rapid pathogen detection within a short period of time. In recent years, an increasing number of scholars combined RAA with CRISPR to increase the detection limit of the assay. RAA and CRISPR-Cas13a’s detection methods have been successfully used to detect swine fever virus (SFV) (Zhang et al., 2022), African swine fever virus (ASFV) (Wei et al., 2022), *Toxoplasma gondii* (Zhao et al., 2022), and porcine epidemic diarrhea virus (PEDV) (Yin et al., 2022).

To establish a faster, more accurate, and more convenient assay to detect H5-AIV, we established a detection system that integrates RAA, CRISPR Cas13a and LFD (RT-RAA-Cas13a-LFD) in this study. We designed nine sets of RAA primers and two specific crRNAs based on the conservative sequence of the H5-AIV HA gene, and reporter RNA with fluorophore-biotin-labeled was synthesized. The assay’s sensitivity and specificity were then assessed. This method offers the advantage of shortening detection time and completing the test at a constant temperature, making it more feasible for farms and grassroots laboratories that have limited resources. Additionally, this method provides a powerful tool for H5-AIV detection.

## Materials and methods

### Virus, clinical samples, and nucleic acid extraction

H3, H5, H7, H9, H10-AIV, infectious bronchitis virus (IBV), Newcastle disease virus (NDV), Group A rotavirus (RVA), and duck astrovirus (DAstV) were provided by the laboratory of the China Animal Health and Epidemiology Center. A total of 380 clinical samples were collected from different provinces (Guangxi, Guangdong, Jiangxi, Hunan, Hubei and Hebei) in China in 2022. The nucleic acids were extracted using FineQuick Rapid Viral DNA/RNA Column Extraction Kit (Genfine Biotech, China).

### Construction of H5-AIV cRNA standard

H5-AIV nucleic acid was amplified by PCR using the primer HA-F/R synthesized by Ruibiotech (Beijing, China). The ligated products were then put into Trans1-T1 phage-resistant chemically competent cells after the PCR products had been gel-purified and cloned into the *pEASY*-T1 vector (TransGen, China), which were spread on Amp-containing LB agar plates for culture. The positive clones were sequenced by Ribbit (Beijing, China). Then, the H5-AIV standard recombinant plasmid was extracted using the TIANperp Mini Plasmid Kit (TIANGEN, China). The extracted plasmid was enzymatically digested and identified. Following this, the plasmid was transcribed *in vitro* by the HiScribe® T7 High Yield RNA Synthesis Kit (NEB, America). Finally, the concentration of the standard was determined using the Multiskan GO (Thermo Fisher, China).

### Design of primers, crRNA, and reporter RNA

The RAA primers specific to the HA gene were constructed by aligning sequences for each gene from GenBank and targeting conserved regions within these H5-AIV sequences. The T7 RNA polymerase promoter sequence (T7) (GAAATTAATACGACTCACTATAGGG) was present in the 5′-primers. Additionally, a highly conserved area of the H5-AIV HA gene was searched for the crRNA, included in the primer amplification region and consisting of the LwaCas13a fixed neck-loop structure and a target sequence of 28 nt in length. Then, the Weblogo website^2^ *was used for* evaluating the conservation. Table 1 displays the sequence of the reporter RNA, which was designed based on the work of Tian et al. (2023). Both the primers and crRNA probe were synthesized by Sangon Biotech (Shanghai, China). All nucleic acid sequences used in this study are shown in Table 1.

**Table 1:** Sequence of primers, crRNA, and Reporter RNA.

Name	Sequence(5′-3′)
HA-F	GGGAGCAAAAGCAGGGG
HA-R	GGAGTAGAAACAAGGGTGTTTT
RT-RAA-F1	AGAACTCTAGATTTCCATGACTCAAATGT
RT-RAA-F2	TTCCATGACTCAAATGTCAAGAACCTTTAT
RT-RAA-F3	TTATGACAAAGTCCGACTACAGCTTAGG
RT-RAA-R1	CCTTTTTAATCTTGCTTCTTCTGAGTACTG
RT-RAA-R2	AATCTTGCTTCTTCTGAGTACTGGGGGTAG
RT-RAA-R3	CTTGCTTCTTCTGAGTACTGGGGGTAGT
T7-RT-RAA-F3	*GAAATTAATACGACTCACTATAGGG*TTATGACAAAGTCCGACTACAGCTTAGG
crRNA1	**GGGAUUUAGACUACCCCAAAAACGAAGGGGACUAAAAC**UCGAAACAACCAUUACCCAGCUCCUUUG
crRNA2	**GGGAUUUAGACUACCCCAAAAACGAAGGGGACUAAAAC**ACAACCAUUACCCAGCUCCUUUGCAUUA
Reporter RNA-LFD	5′/6-FAM/UUUUUUUUUUUUUUUUUUUU-Biotin/3′
Reporter RNA-BHQ	5′/6-FAM/UUUUUUUUUUUUUUUUUUUU-Biotin/3′

Italicized sections represent T7 promoter sequences; bold sections represent the fixed neck loop structure of LwaCas13a.

RT-RAA reactions were performed according to the instructions of the RT-RAA basic kit (ZC Bioscience, China). Each RAA reaction was conducted in a reaction volume of 50 μL, comprising 25 μL of buffer A, 2 μL forward and reverse primers (10 μM), 13.5 μL RNase-free water, 5 μL target RNA template, and 2.5 μL buffer B added to the tube caps. After being vortexed and rotated briefly, the reaction tubes were placed in a NaCha Pro multi-temperature zone metal bath (Monadbiotech, China) at 37°C for 30 min.

### Establishment of the RT-RAA-Cas13a-LFD assay

The RT-RAA-Cas13a-LFD reaction systems has a volume of 50 μL, consisting of rNTP Mix (25 mM/each, 4 μL) (New England Biolabs), RNase inhibitor (40 IU/μL, 2 μL) (Vazyme, China), LwaCas13a nuclease (100 ng/μL, 4 μL) (Genscript, China), T7 polymerase (50 IU/μL, 1 μL) (NEB, America), Mgcl_2_ (1 M, 0.5 μL) (Sangon, China), HEPES (1 M, 1 μL) (Sangon, China), crRNA (33.4 ng/μL, 3 μL), lateral flow reporter RNA (100 nM, 5 μL), RNase-free water (26.5 μL) (Vazyme, China), and 5 μL of RT-RAA amplification products. This mixture was thoroughly mixed and incubated at 37°C for 20 min. Subsequently, 50 μL of the reaction products were added to the LFD, and the results were obtained within 3–5 min, followed by capturing pictures.

### Interpretation of RT-RAA-Cas13a-LFD assay result

**Figure 1:**
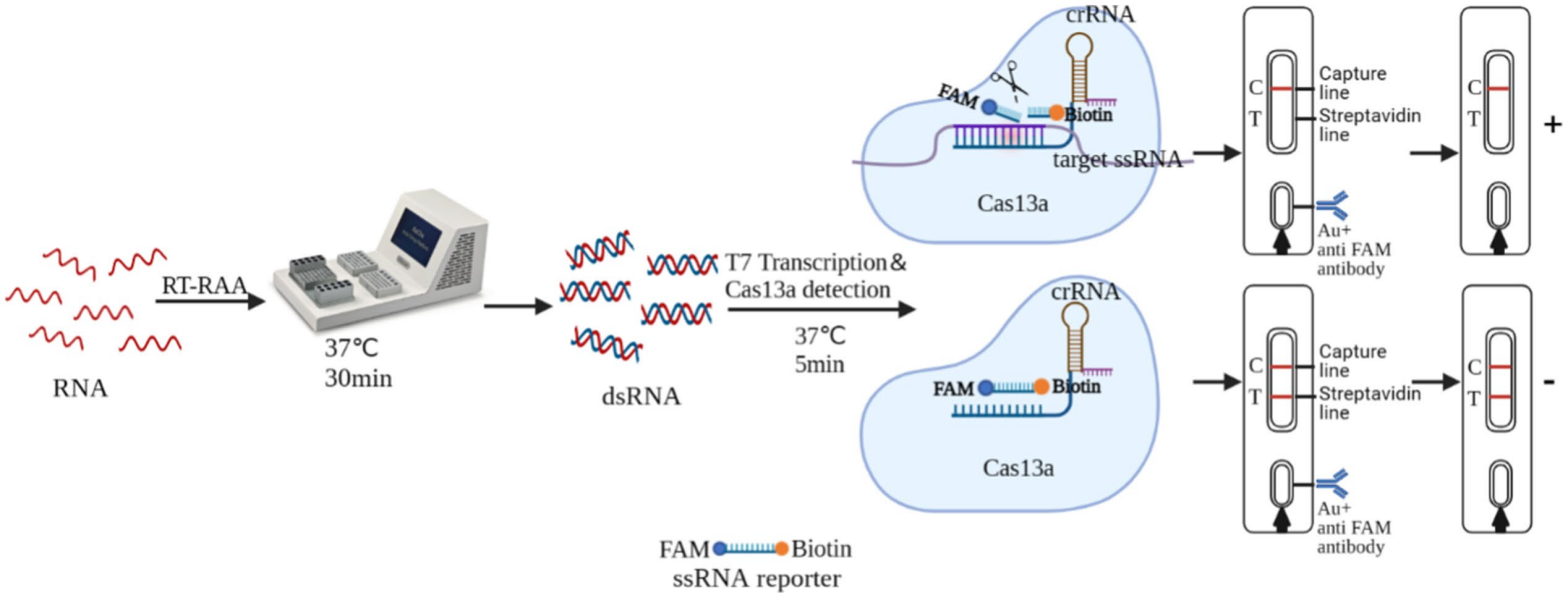
Schematic of the RT-RAA-Cas13a-LFD detection assay for H5-AIV.

RT-RAA-Cas13a-LFD is based on the trans-cleavage activity of the Cas13a protein. The sample pad of the LFD was coated with colloidal gold-labeled rabbit-derived anti-FITC antibody. The T-line was labeled with streptavidin, while the C-line was labeled with goat anti-rabbit or mice IgG antibody. The complex binds to the sample to be tested, activating the non-specific RNase activity of the Cas13a protein. This resulted in “cutting” the reporter RNA with the FAM and Biotin attached to it. When the reaction mixture passed through the sample pad of the LFD, the FAM end of the reporter RNA bound to the colloidal gold-labeled antibody, forming “FAM-anti-FITC antibody-colloidal gold” complex. This complex does not bind to the streptavidin when passing through the T line, and as a result, no bands appear in the T-line. However, it was captured by the C line antibody, causing the colloidal gold to deposit and yielding a positive result on the C line. If the system does not contain the target nucleic acid, the activity of the Cas13a cleavage reporter RNA will not be activated. When the reaction mixture passed through the sample pad of LFD, the FAM end of the reporter RNA bound to the colloidal gold-labeled rabbit-derived anti-FITC antibody, forming “biotin-FAM-anti-FITC antibody-colloidal gold” polymer. The polymer bound to the streptavidin on the T-line as it passed through the T-line and the colloidal gold settles on the T line, and this was captured by the C-line antibody as it passes through the C-line and the colloidal gold collected on the C-line, yielding a negative result (Figure 1).

### Optimizing of the RT-RAA-Cas13a-LFD reactions

The reporter RNA concentration, Cas13a concentration and their reaction time may affect the amplification efficiency of the RT-RAA-Cas13a-LFD assay. First, the concentration of the optimized reporter RNA was set to 5 nM, 10 nM, and 20 nM. Then, the remaining factors were kept constant. The Cas13a concentration was set to 50, 100, 250 and 500 ng/μL under the optimal reporter RNA concentration, and the remaining factors were kept constant. Finally, the reaction time was set to 5 min, 10 min, and 15 min under the optimal reporter RNA and Cas13a concentration conditions.

### Specificity and sensitivity test of the RT-RAA-Cas13a-LFD assay

Poultry virus nucleic acid samples, such as H3, H7, H9, H10-AIV, IBV, NDV, RVA and DAstV were used to assess the specificity of the RT-RAA-Cas13a-LFD assay established in this study. Then, RNase-free water was added to the reaction system as a negative control.

The RT-RAA-Cas13a-LFD assay’s sensitivity was evaluated using the serially diluted H5-AIV cRNA standard (10^6^–10^−2^ copies/μL), which were prepared and amplified using the RT-RAA assay. The resulting reaction products were then used to analyze the sensitivity of the CRISPR-Cas13a-LFD.

### Sensitivity comparison of RT-qPCR and RT-RAA-Cas13a-LFD assay

The same concentration of the H5-AIV cRNA standard was used as a template for the RT-qPCR reaction system, which was compared with the RT-RAA-Cas13a-LFD method. The RT-qPCR reaction system conducted in a reaction volume of 20 μL, comprising 10 μL of One Step RT-qPCR Buffer II (Probe) (2X) (Agbio, China), 1 μL of forward and reverse primers (10 μM), 0.5 μL of the probe reporter (2 μM), 0.4 μL of Pro Taq HS DNA Polymerase, 0.4 μL of Evo M-MLV RTase Enzyme Mix II, 2 μL of RNA templates, and 4.7 μL of RNase-free water. The assays were performed using the QuantStudio™ 5 Pro Real-Time PCR System (Thermo Fisher, China), which involved denaturation at 42°C for 5 min; 95°C for 30 s; 95°C for 5 s, and 60°C for 30 s, with a total of 40 cycles. There was also a negative control in which the RNA template was swapped out for RNase-free water.

### Repeatability of RT-RAA-Cas13a-LFD assay

To determine the repeatability of the RT-RAA-Cas13a-LFD assay, three different concentrations of H5-AIV cRNA standards were used and amplified at different periods by the RT-RAA assay. Furthermore, these reaction products were used to examine the repeatability of the CRISPR-Cas13a-LFD. As a negative control, RNase-free water was added to the reaction system.

### Evaluation of the detection of RT-RAA-Cas13a-LFD assay

There were a total of 380 clinical samples submitted for the detection of H5-AIV using RT-RAA-Cas13a-LFD to analyze and evaluate the feasibility of applying this method in clinical samples. The same samples were also detected using the RT-qPCR method recommended by the WOAH, and the results of the two assays were compared and analyzed. The consistency of these two methods was compared using SPSS 20.0 software. κ-tests were used for consistency analysis.

## Results

### Screening of primers for H5-AIV detection by the RT-RAA

**Figure 2:**
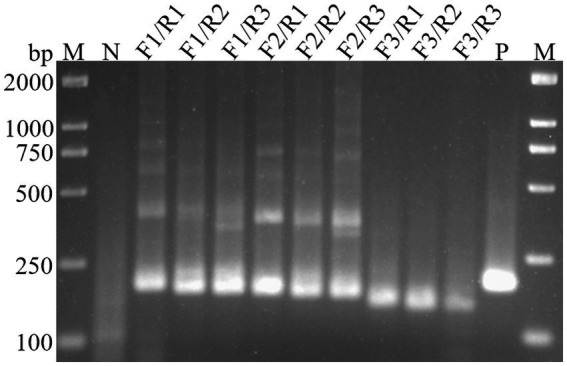
Screening of primers for RT-RAA assay. M, DL2000 DNA Marker; N, Negative control; P, Positive control.

To identify the most efficient primer set for the RT-RAA-Cas13a-LFD method, the H5-AIV HA gene sequence was downloaded from NCBI. Using Oligo 7 software, three upstream and three downstream primers were designed to target highly conserved regions. These primers were then combined into nine pairs, which were amplified by RT-RAA and analyzed using 2% agarose gel electrophoresis. The results showed that the primer pairs, F3/R1, F3/R2, and F3/R3, exhibited no significant non-specific amplification. Moreover, the amplification efficiency of F3/R1 was higher than the other two pairs (Figure 2). Therefore, the F3/R1 combination was used as the primer pair for subsequent experiments.

### Screening of the optimal crRNA sequence

**Figure 3:**
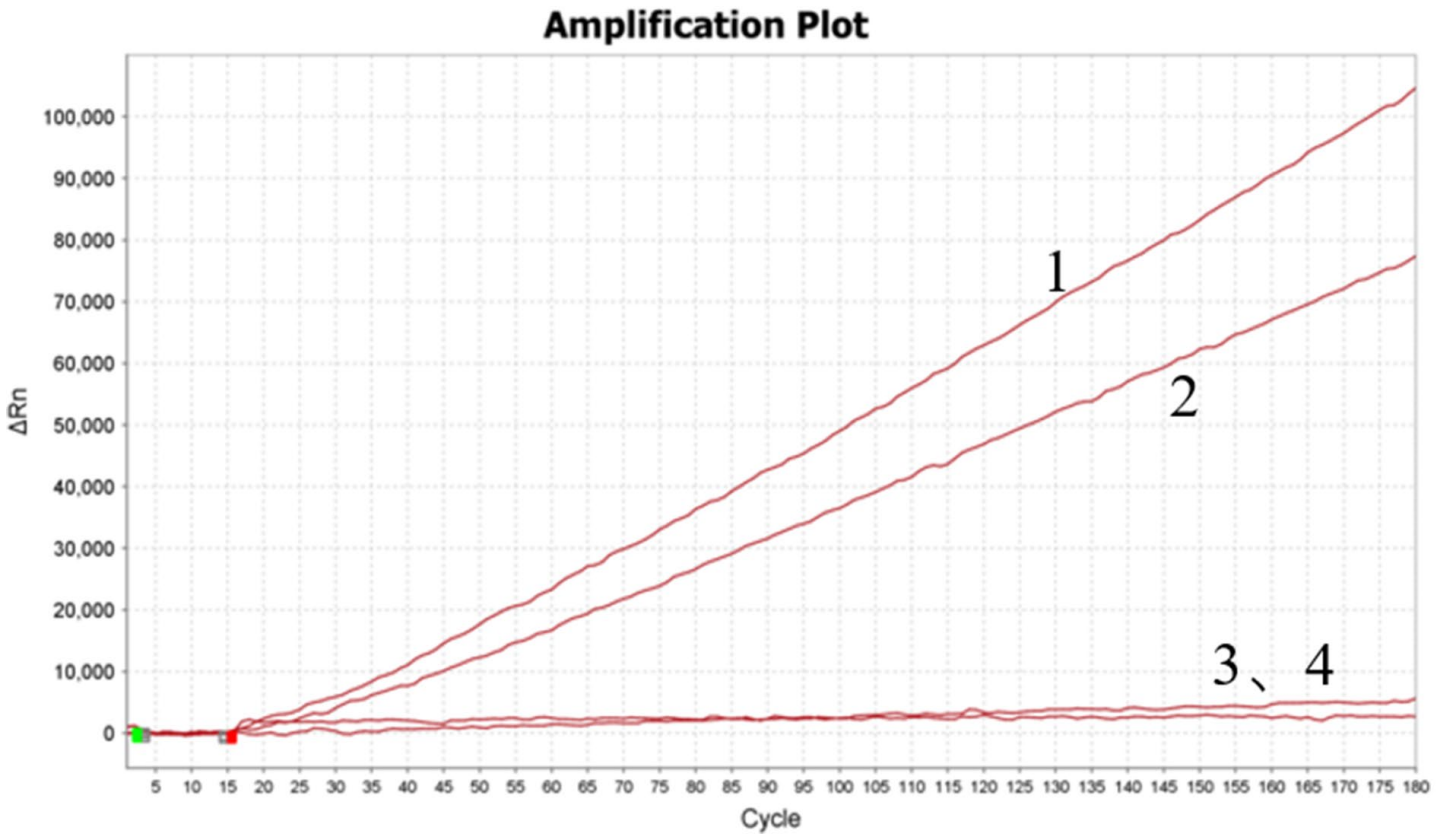
Screening of designed crRNAs using QuantStudio™ 5 Real-Time PCR System. 1–4: crRNA1; crRNA2; crRNA1 negative control; crRNA2 negative control.

**Figure 4:**
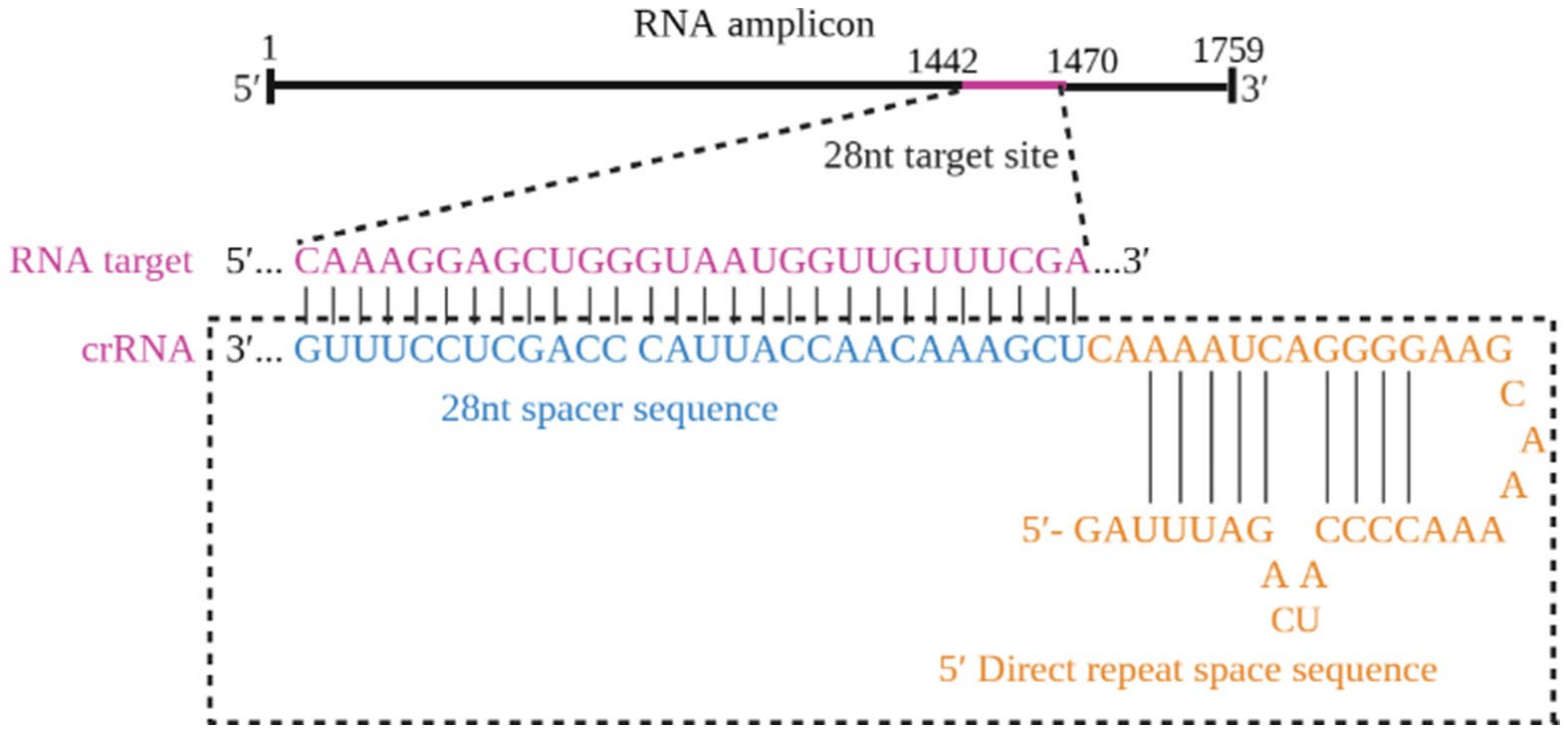
The specific design scheme of crRNA.

**Figure 5:**
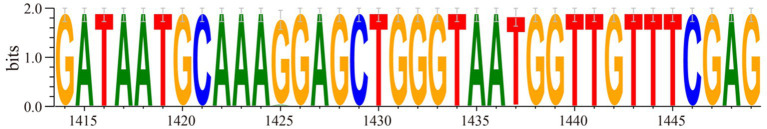
Conserved analysis of crRNA1.

The optimized crRNA sequence for the RT-RAA-Cas13a-LFD system was evaluated by the screening of designed crRNAs using the QuantStudio™ 5 real-Time PCR system (ThermoFisher, China). For the RT-RAA-Cas13a-BHQ reaction, 3 μL of crRNA (33.4 ng/μL) was added and the mixture was incubated at 37°C 1 min; 37°C 10 s, 180 cycles. The results indicated that crRNA1 fluorescence signal value was higher than that of crRNA2. Thus, crRNA1 was selected for the RT-RAA-Cas13a-LFD assay system (Figure 3). The reverse complementary sequence of this target sequence is combined with the 38 bp LwaCas13a protein-bounding anchor sequence (5’-GGGAUUUAGACUACCCCAAAAACGAAGGGGACUAAAAC-3′) to form crRNA. The specific design scheme of crRNA is shown in Figure 4. The conservation of crRNA1 was analyzed using Weblogo, and each locus in the sequence identity map has a dominant nucleotide, indicating that the crRNA sequence is highly conserved (Figure 5).

### Optimizing of the RT-RAA-Cas13a-LFD reactions

**Figure 6:**
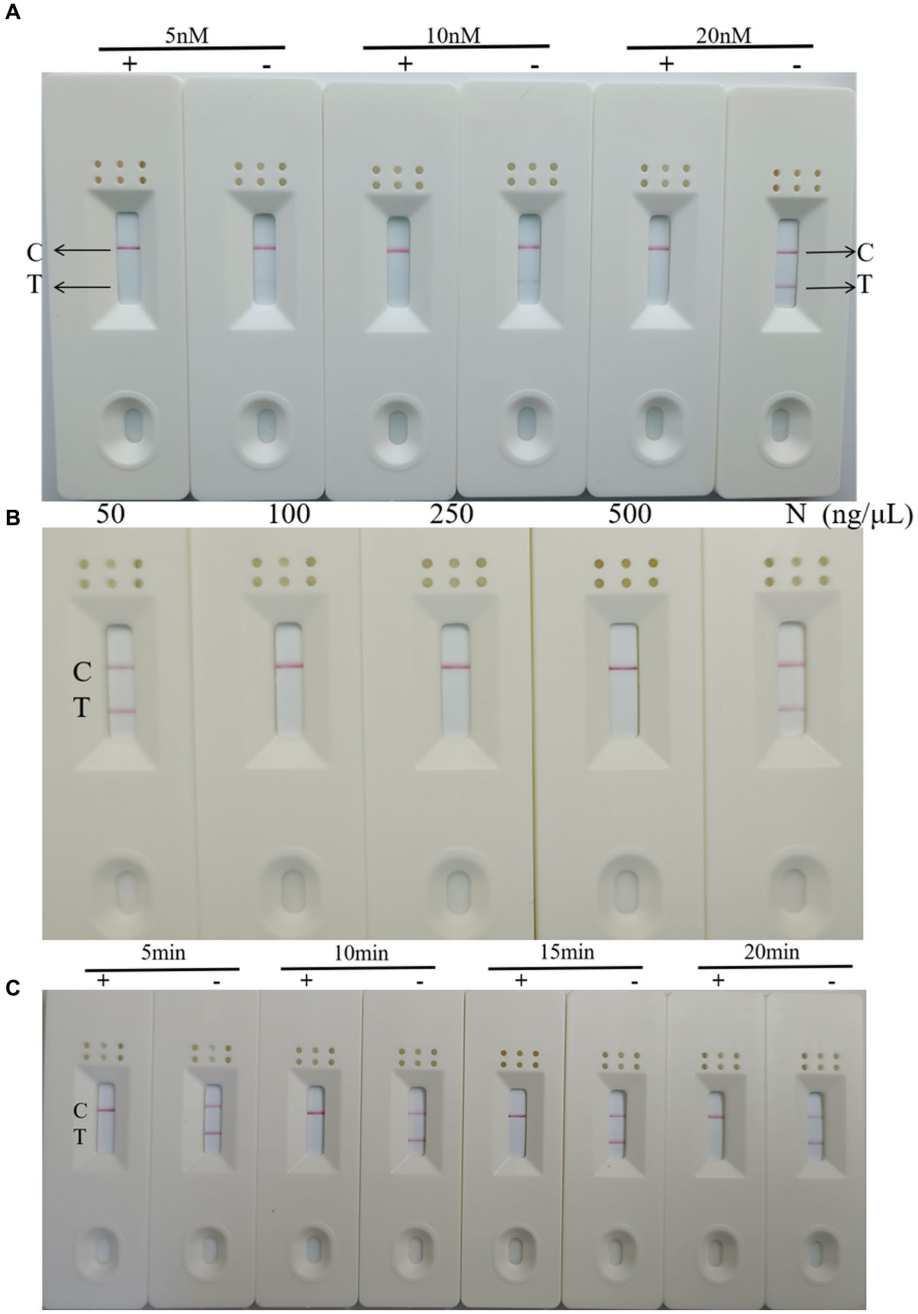
Optimization of RT-RAA-CRISPR Cas13a-LFD reaction conditions **(A)** Optimization of reporter RNA concentration. C: quality control line; T: test line; +: positive result; −: negative result. **(B)** Optimization of Cas13a concentration. C: quality control line; T: test line. **(C)** Optimization of reaction time. C: quality control line; T: test line; +: positive result; −: negative result.

To optimize the concentration of the reporter RNA, RT-RAA-Cas13a-LFD reactions were conducted at 5 nM, 10 nM, and 20 nM at 37°C for 20 min. The results showed that C-lines appeared in the positive samples when the concentrations of the reporter gene RNA were 5, 10, and 20 nM, while the negative control T-lines showed only weak bands at 5 nM and 10 nM, becoming darker at 20 nM (Figure 6A). Therefore, a concentration of 20 nM was used as the best reporter RNA in the experiment.

To optimize the concentration of Cas13a, RT-RAA-Cas13a-LFD reactions were performed at 50, 100, 250, and 500 ng/μL at the optimal reaction time and reporter RNA concentration. The results showed that the LFD results were negative when the Cas13a concentration was 50 ng/μL, whereas the results were positive at concentrations of 100, 250, and 500 ng/μL (Figure 6B). Therefore, Cas13a at a concentration of 50 ng/μL was used as the optimal concentration for this experiment.

Finally, on the basis of these results, RT-RAA-Cas13a-LFD reactions were performed for 5 min, 10 min, 15 min, and 20 min to determine the reaction time. The result indicated that the target template was successfully amplified, and when the reaction time was extended, the band color of the negative and positive samples does not change significantly (Figure 6C). Considering the accuracy of the results, 10 min was identified as the optimal reaction time for this method. In summary, the RT-RAA-CRISPR Cas13a assay requires 30 min for RAA amplification and 10 min for CRISPR Cas13a detection, for a total of 40 min. This is less than the 1–2 h required for RT-qPCR and RT-PCR.

### Specificity and sensitivity of the RT-RAA-Cas13a-LFD assay

**Figure 7:**
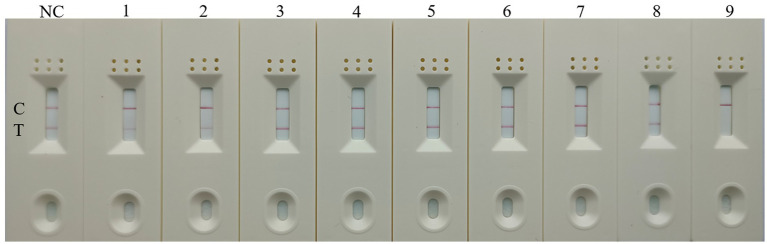
Specificity of the RT-RAA-CRISPR Cas13a-LFD assay. 1–9: H3, H7, H9, H10-AIV, IBV, NDV, RVA, DAstV; H5-AIV; C: quality control line; T: test line.

To evaluate the specificity of RT-RAA-Cas13a-LFD assay, other kinds of poultry pathogens were selected for detection, including H3, H7, H9, H10-AIV, IBV, NDV, RVA, and DAstV. H5-AIV was selected as the positive control and RNase-free water served as the negative control. The combination of primer set 1, a reaction time of 10min, and a crRNA concentration of 20 nM combination was selected for detection. The result showed that only the LFD with H5-AIV as a template was positive. Other significant poultry viruses, including H3, H7, H9, H10-AIV, IBV, NDV, RVA, and DAstV displayed no visual positivity in the negative control (Figure 7). This demonstrates that the established RT-RAA-Cas13a-LFD assay exhibits good specificity for detecting H5-AIV.

**Figure 8:**
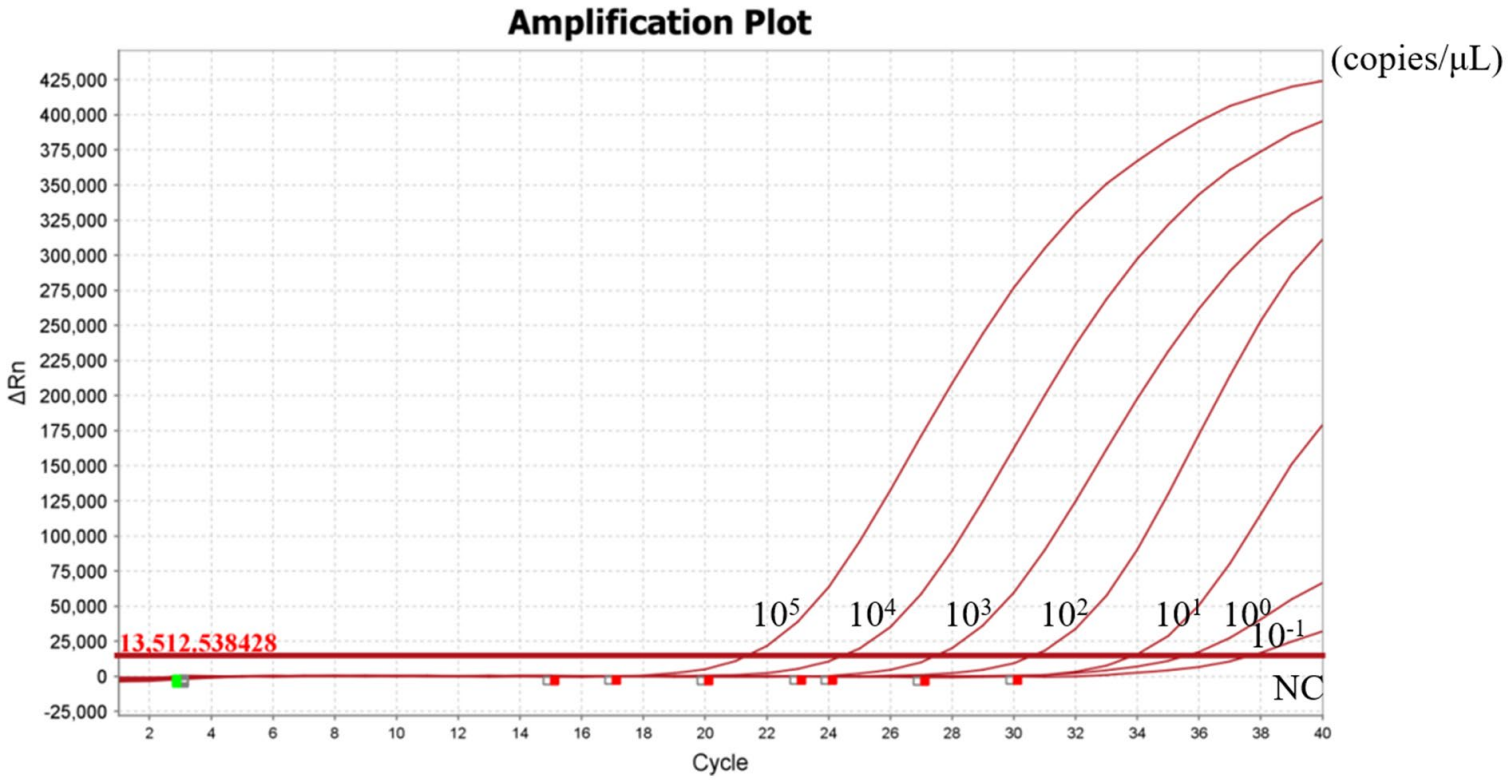
Sensitivity of RT-qPCR assay. NC: Negative control.

**Figure 9:**
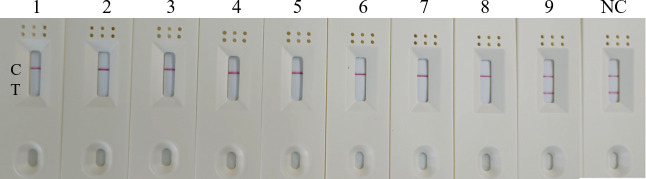
Sensitivity of the RT-RAA-CRISPR Cas13a-LFD assay. NC: Negative control.

To evaluate the sensitivity of the RT-RAA-Cas13a-LFD assay, a series of 10-fold gradient dilutions of H5-AIV cRNA were used as the template for the RT-RAA-Cas13a-LFD assay. These dilutions ranged from 10^6^ copies/μL to 10^−2^ copies/μL, with RNase-free water included as a negative control. Based on the LFD result, the control lines were visible when the concentration of the H5-AIV cRNA standard was more than >10^−1^ copies/μL (Figure 8). Further verification by RT-qPCR showed that the minimum detection limit of RT-qPCR was 10^−1^ copies/μL (Figure 9). This result indicates that the detection limits of the two methods were consistent.

### Repeatability of RT-RAA-Cas13a-LFD assay

**Figure 10:**
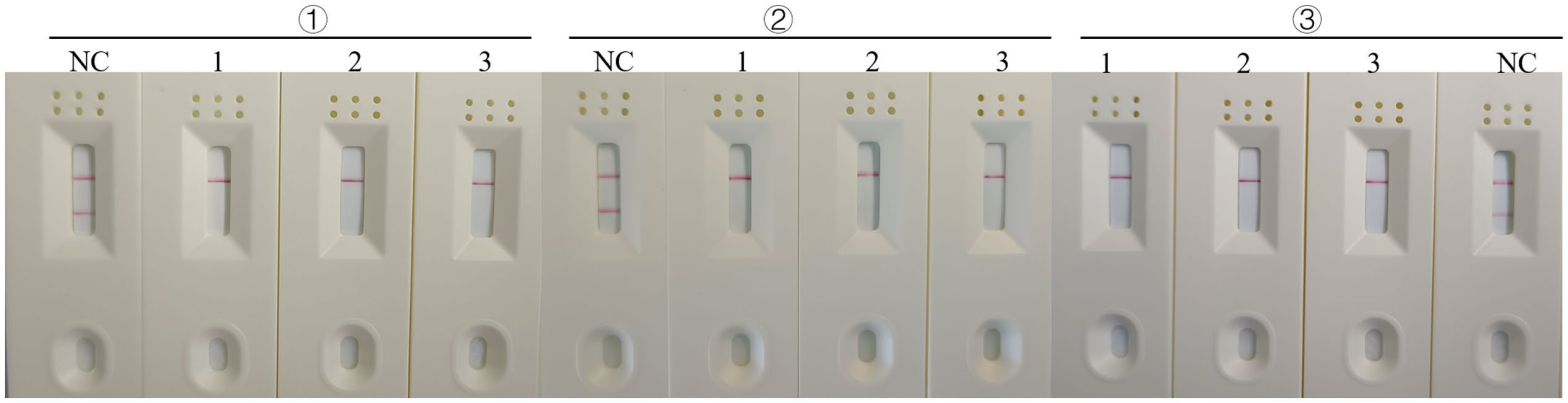
Repeatability of the RT-RAA-CRISPR Cas13a assay. 1:10^6^ copies/μL; 2: 10^3^ copies/μL; 3:10^0^ copies/μL; NC: Negative control.

In addition to establishing the test’s analytical sensitivity and specificity, the RT-RAA-Cas13a-LFD assays was validated for repeatability. The detection was performed at different time points using 10^6^, 10^3^, and 10^0^ copies/μL on H5-AIV cRNA standards. The results showed that positive samples with high, medium, and low concentrations could be detected at three different time points (Figure 10). The preceding date indicates that the established RT-RAA-Cas13a-LFD has good repeatability in detecting H5-AIV.

### Evaluation of the detection of RT-RAA-Cas13a-LFD assay

To verify the reliability of RT-RAA-Cas13a-LFD in the detection of H5-AIV clinical sample, a total of 380 clinical samples were collected from Guangxi, Guangdong, Jiangxi, and other Chinese provinces in 2022. The collected samples were tested by RT-qPCR. As shown in Table 2, 38 samples were detected as H5-AIV positive by RT-qPCR method. The RT-RAA-Cas13a-LFD assay detected 31 H5-AIV positive samples and 349 negative samples. The assay’s results were in good agreement (*κ* = 0.89) with those from the RT-qPCR results (Table 2).

**Table 2:** Comparison of RT-RAA-Cas13a-LFD with RT-qPCR for detection of the clinical samples.

		RT-qPCR		Total	Sensitivity	Specificity	Kappa Value
		+	−				
RT-RAA-Cas13a-LFD	+	31	0	31	81.6%	100%	0.89
	−	7	342	349			
	Total	38	342	380			

+, positive result; −: negative result.

## Discussion

The H5 subtype of HPAI poses a serious threat to the global poultry industry, and as a zoonotic virus, it has become a focus of human concern (Li et al., 2021). The spread of the zoonotic H5N1 virus in poultry has created a risk of transmission to humans, making it a significant threat to human public health (He et al., 2020). RT-qPCR is recognized as one of the official methods recommended by the WOAH to confirm the presence of the virus in laboratory samples (Auer et al., 2023), since it must require sophisticated instrumentation and operator expertise and is not suitable for rapid on-site diagnosis. Therefore, there is an urgent need for a portable, rapid, and accurately tested diagnostic method.

The CRISPR/Cas system was first developed for the diagnosis of molecular nucleic acids in 2016 (Pardee et al., 2016). CRISPR diagnostics frequently rely on the pre-amplification of target nucleic acids to increase detection sensitivity, followed by the combination of Cas proteins for detection. RAA can amplify viral nucleic acids at 37–42°C without the need for special temperature-changing instruments. Moreover, it has been used in combination to improve the sensitivity of CRISPR/Cas assays. With the increasing demand for instrument-free nucleic acid detection technologies, CRISPR/Cas systems have been combined with a variety of isothermal amplification technologies, such as RAA, RPA, LAMP, and ERA, and the detection results are displayed in a various formats that can be read by fluorescent signals, colorimetric signals, electrochemical signals, and LFD techniques (Yin et al., 2021). *In vitro* diagnostic platforms based on the CRISPR/Cas system have been used to detect various types of pathogens, including bacterial diseases (Zhou et al., 2020; Schultzhaus et al., 2021), viral diseases (Barnes et al., 2020; Fozouni et al., 2021; Hu et al., 2022; Tian et al., 2023), parasites (Zhao et al., 2022; MacGregor et al., 2023), and tumors (Li et al., 2022). Among them, the most famous SHERLOCK and DETECTOR as two new nucleic acid detection technologies can maintain a constant temperature of 37°C throughout the whole process. The detection time is shorter than the RT-qPCR method, and the results can be read by the naked eye, combining the high sensitivity and specificity brought by Cas protein, which is a powerful weapon to cope with the emerging and sudden major infectious diseases around the world.

In this study, we established a new H5-AIV assay combining RT-RAA, CRISPR-Cas13a and LFD (RT-RAA-Cas13a-LFD). We designed primers for RT-RAA, based on the highly conserved fragment of the H5-AIV HA gene, and then searched for and synthesized crRNA sequences in the middle of the primers. After the screening of the best primers and crRNAs, we optimized the reaction conditions. The F3/R1 primer combination, the reaction temperature of 37°C, the reporter RNA concentration of 20 nM, and the Cas13a concentration of 100 ng/μL were determined as the optimal reaction conditions for this method, thus achieving the rapid and accurate detection of H5-AIV in the field. The assay was specific and showed no cross-reactivity with common avian pathogens. The cRNA standard could be detected at 0.1 copy/μL, which is consistent with the sensitivity of RT-qPCR. In addition, high, medium, and low concentrations (10^6^, 10^3^ and 10^0^ copies/μL) of H5-AIV cRNA standards were detected at three different times, indicating that the method has good reproducibility. The RT-RAA-Cas13a-LFD and RT-qPCR methods showed 98.16% agreement and a kappa value of 0.89 (*κ* > 0.75) for the same samples, and the results of the two assays were in high agreement with each other. Although RT-qPCR has a high detection rate for H5-AIV samples, RT-RAA-Cas13a-LFD is not labor-intensive or time-consuming, and is more suitable for field testing.

RT-RAA-Cas13a-LFD does not require the use of complex instruments, which not only shortens the detection time but also simplifies the detection process and enables visualization of the results. Therefore, we believe that RT-RAA-Cas13a-LFD is a powerful tool for in-field detection of H5-AIV. Despite the above advantages of the method, this study was a two-part assay with the risk of repeated opening and contamination. In the future study, we hope to perform RT-RAA and CRISPR in a single reaction to further reduce the operational steps and reaction time.

In summary, we developed RT-RAA-Cas13a-LFD for rapid field diagnosis of H5-AIV, which can be used for rapid, sensitive, and portable detection of H5-AIV. It is important for timely detection and control of H5-AIV outbreaks, avoiding the threat posed by H5-AIV to public health security.

## Data availability statement

The original contributions presented in the study are included in the article/Supplementary material, further inquiries can be directed to the corresponding author.

## Ethics statement

The animal studies were approved by Animal Welfare Committee of the China Animal Health and Epidemiology Center; It is attributed to China Animal Health and Epidemiology Center. The studies were conducted in accordance with the local legislation and institutional requirements. Written informed consent was obtained from the owners for the participation of their animals in this study.

## Author contributions

YL: Project administration, Supervision, Writing – original draft, Writing – review & editing. JS: Data curation, Methodology, Writing – original draft. JL: Data curation, Investigation, Methodology, Writing – original draft. FZ: Data curation, Methodology, Writing – original draft. GM: Methodology, Writing – original draft. YF: Methodology, Writing – original draft. WJ: Resources, Supervision, Writing – original draft. XY: Resources, Supervision, Writing – original draft. CD: Methodology, Writing – original draft. GL: Investigation, Project administration, Supervision, Writing – review & editing. HL: Formal analysis, Investigation, Project administration, Supervision, Writing – review & editing.

## Footnotes

^1^ https://www.woah.org/en/home/

^2^ https://weblogo.threeplusone.com/manual.html
