# Supplementary figures and images for: Rapid detection of H5 subtype avian influenza virus using CRISPR Cas13a based-lateral flow dipstick
Source: Front Microbiol. 2023 Nov 29;14:1283210. doi: 10.3389/fmicb.2023.1283210 (PMC10716353; doi:10.3389/fmicb.2023.1283210)

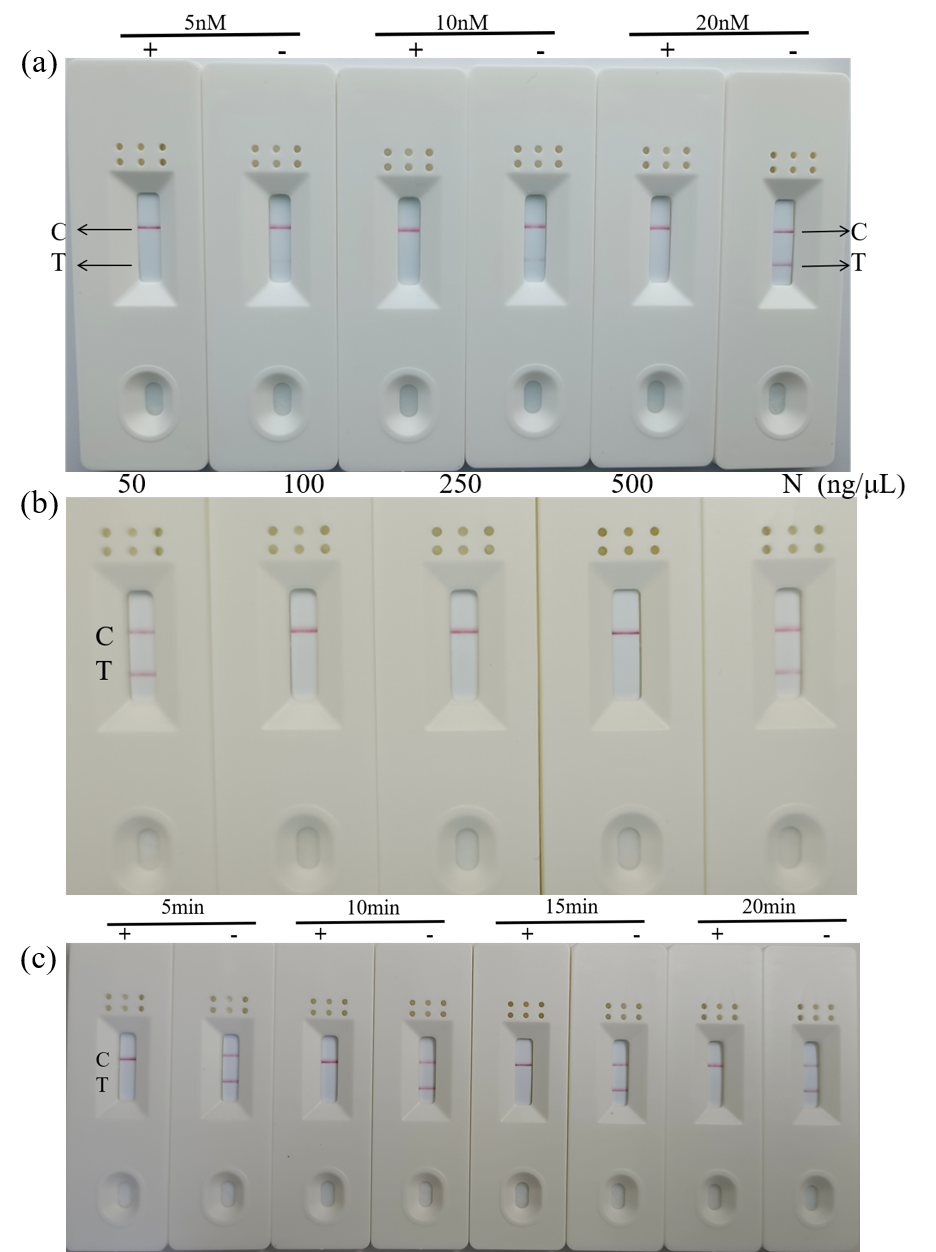

Supplement: Supplementary file 1 [file Image_1.TIFF]

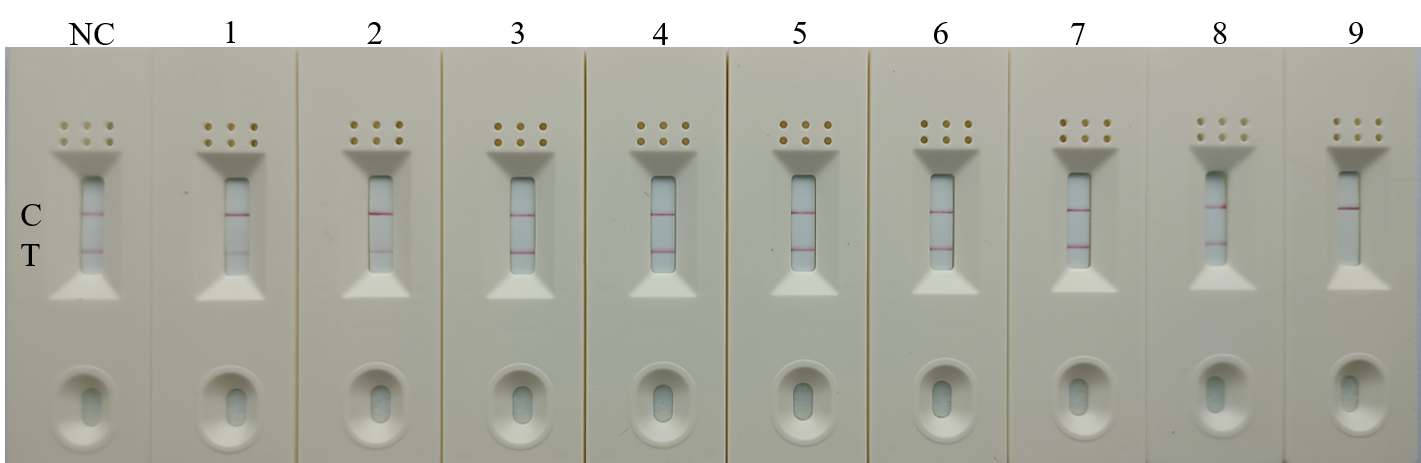

Supplement: Supplementary file 2 [file Image_2.TIFF]

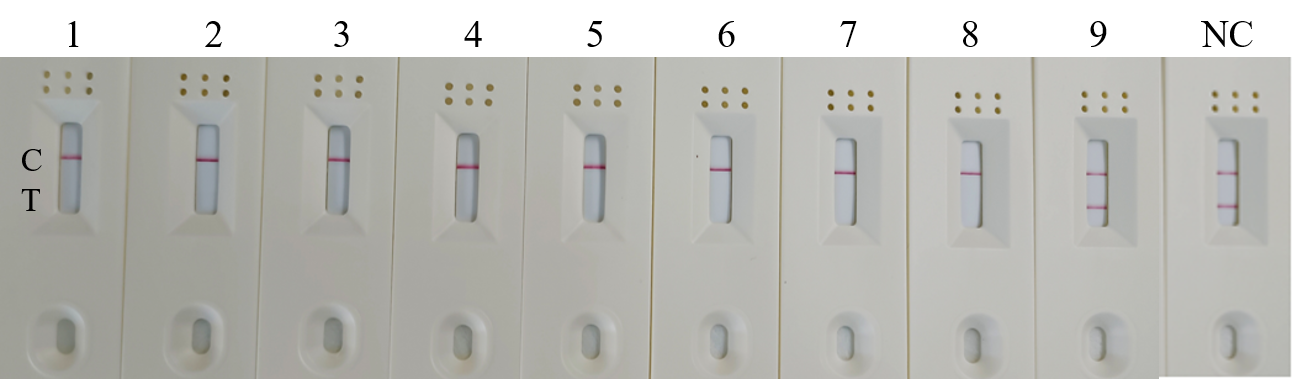

Supplement: Supplementary file 3 [file Image_3.TIFF]

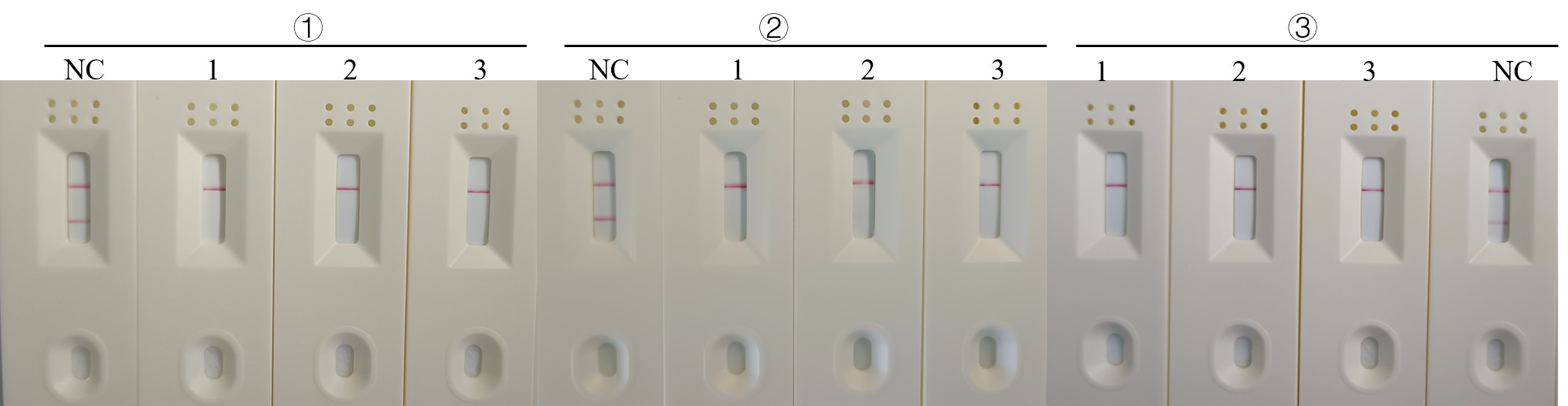

Supplement: Supplementary file 4 [file Image_4.TIFF]
